# Trends in HIV Prevalence and HIV-Related Risk Behaviors Among Male Students Who Have Sex With Men From 2016 to 2020 in Nanjing, China: Consecutive Cross-Sectional Surveys

**DOI:** 10.3389/fpubh.2022.806600

**Published:** 2022-04-27

**Authors:** Yuanyuan Xu, Sushu Wu, Xuezhen Fu, Jie Ding, Wenjiong Xu, Xin Li, Hongjie Shi, Mengkai Qiao, Zhengping Zhu

**Affiliations:** ^1^Department of AIDS/STD Control and Prevention, Nanjing Center for Disease Control and Prevention, Nanjing, China; ^2^International Department of Nanjing No.13 Middle School, Nanjing, China; ^3^Department of Office, Nanjing Center for Disease Control and Prevention, Nanjing, China; ^4^Department of Microbiology Laboratory, Nanjing Center for Disease Control and Prevention, Nanjing, China

**Keywords:** HIV/AIDS, men who have sex with men, prevalence, risk behaviors, students

## Abstract

**Background:**

The growing HIV epidemic among student men who have sex with men (MSM) necessitates immediate attention from public health. In China, male students who have sex with men (SMSM) were also at an increasing risk of HIV transmission. The aim of this study was to investigate the trends in HIV prevalence, HIV-related risk behaviors, and HIV testing, as well as analyze the factors associated with HIV infection among SMSM in Nanjing.

**Methods:**

Data were collected through face-to-face questionnaire interviews and laboratory testing in Nanjing. The participants were recruited among SMSM by snowball sampling and internet-recruited convenience sampling annually from 2016 to 2020. The self-report data primarily included demographics, HIV knowledge, HIV-related behaviors, and HIV testing, while the laboratory test results of HIV and syphilis were collected. Linear-by-linear chi-square test was used to analyze the trends of HIV/syphilis prevalence and its risk behaviors. The binary logistic regression model was used to explore the factors associated with HIV infection.

**Results:**

During the 5 years from 2016 to 2020, a total of 775 SMSM were recruited in our survey (220, 112, 171, 142, and 120, respectively). The HIV prevalence was 5.2, 6.3, 5.3, 7.0, and 8.3%, without a significant increasing trend (*P* = 0.277). Syphilis prevalence fluctuated from 5.7% in 2016 to 4.2% in 2020, without a significant decreasing trend (*P* = 0.318). The proportion of consistent condom use in anal intercourse (48.5 to 56.2%, *P* < 0.05), and HIV testing in the past 12 months (51.0 to 59.2%, *P* < 0.05) were increasing. A remarkable growing trend has been reported in the percentage of MSM with more than one male sex partner (46.2 to 59.2%, *P* < 0.05). Multivariate analysis showed that HIV testing in the past 12 months was a protective factor against HIV infection. MSM who had unprotected anal intercourse (UAI) in the past 6 months, recreational drug use, and currently syphilis infection were risk factors for HIV infection.

**Conclusions:**

We observed stable HIV/ syphilis prevalence, increasing consistent condom use, increasing HIV testing rate, and increasing multiple male sex partners dramatically among SMSM in China. The original comprehensive intervention measures should be continuously strengthened for the subgroup. To satisfy the current HIV prevention requirements, new biological interventions should be introduced and carried out as major components of combination prevention programs.

## Introduction

Human immunodeficiency virus (HIV) epidemic among MSM has aroused nationwide substantial concern. The overall HIV prevalence among MSM in China expanded steadily from 5.5% in 2009 (1) to 8.0% in 2015 (2). A meta-analysis extracted from 355 cross-sectional studies and covering 59 cities of China also displayed a climbing tendency in the HIV prevalence among MSM from 2001 to 2018 (3). SMSM (also male students who have oral or anal sex with men) is a unique subgroup of the MSM community, regardless of their sexual orientation or sexual identity, facing an enlarging risk of HIV transmission in China. The newly diagnosed student HIV cases increased from 794 cases in 2010 to 3422 cases in 2019 in China. The 10 years' surveillance (2010–2019) described a total of 23,307 newly-diagnosed student HIV cases, 80.0% of which were infected via homosexual transmission (4). A multicenter cross-sectional study in seven major Chinese cities showed that the HIV incidence rate were 10.0/100 person-year among college student MSM during 2012–2013 (5). Recent meta-analyses also showed that the prevalence of HIV infection among SMSM in China increased from 3.0% between 2003 and 2006 (6) to 5.2% between 2012 and 2016 (7). This rising HIV prevalence among SMSM identifies a distinct risk population that could have an influence on HIV transmission in the school.

Previous studies indicated that HIV prevalence among MSM in Nanjing remained steady at a high level from 2013 to 2017 (8). Among the 183 newly diagnosed student HIV cases reported from 2002 to 2014, 90.2% were infected via male homosexual transmission (9). The newly diagnosed student HIV cases from 2011 to 2016 were 20, 36, 42, 80, and 86, respectively and the proportion of homosexual transmission reached to 93.4% (10). In a previous study in Nanjing, only 10.9% of HIV-positive student MSM insisted on using condoms when having intercourse with male regular sexual partners and 11.9% when having intercourse with those casual partners (11). Moreover, a previous study showed that HIV prevalence among young MSM in Nanjing were 6.76, 9.91, 10.40, 9.14, and 6.82% from 2013 to 2017, respectively (12). The alarming HIV epidemic among student MSM in Nanjing needs to be handled seriously by public health, as evidenced by the annual growth in the number of newly reported cases and the overwhelming dominance of homosexual transmission.

Nanjing implemented comprehensive prevention strategies to mitigate the HIV epidemic among young students especially SMSM, including acquired immunodeficiency syndrome (AIDS) prevention publicity and education, peer education, condom promotion, voluntary HIV testing and counseling (VCT), immediate antiretroviral therapy (ART), and referrals for standard sexually transmitted diseases (STD) treatment from 2015. *The China Action Plan for the Thirteenth 5-Year Plan for Combating and Prevention of AIDS* were announced by the State Council of China, which outlines the holistic requirements, measures of prevention and treatment, and safeguard measures. The AIDS Prevention and Control Commission of The State Council had launched the third round of national comprehensive demonstration zones for AIDS prevention and control since 2015. Following those requirements, Nanjing city actively explores innovations of HIV prevention for young students in colleges and universities to promote HIV/AIDS prevention and control work, such as strengthening organization management, the regular epidemic notification, multi-department cooperation mechanisms, and expanding the coverage of AIDS education through distinct activities.

However, it is unknown about the effectiveness of these comprehensive strategies among SMSM. Therefore, we conducted a special cross-sectional survey annually. Now, we analyzed the five consecutive surveys from 2016 to 2020 to assess the trends of HIV prevalence, risk behaviors, and HIV testing as well as clarify the factors associated with HIV infection among SMSM.

## Materials and Methods

### Study Settings

The study was carried out in Nanjing, the capital city of Jiangsu Province in eastern China, which has a population of 8.6 million people. There are 44 colleges and universities and 21 vocational schools in Nanjing, resulting in a large number of college students, thus including a sizeable number of SMSM.

### Participants and Recruitment

Male students who had anal or oral intercourse with men in the previous 12 months, were at least 16 years old and were willing to offer written informed consent were included in our questionnaires.

Since the total scale and specific distribution of the SMSM population in Nanjing could not be grasped, the participants were recruited annually from April to July between 2016 and 2020 using snowball sampling and convenience sampling. For the former sampling, volunteers and student volunteers of two MSM Community Based Organizations (CBOs) were mobilized to introduce MSM students who they know to participate in the study. For the latter sampling, we issued recruitment notices through the QQ or WeChat, groups of the MSM CBOs and the Nanjing CDC official website, recruiting SMSMs who are willing to participate.

### Data Collection

The surveys were conducted at the VCT clinics of Nanjing CDC or Qinhuai district CDC, whose staff members were annually well-trained interviewers and supervisors. Repeated participants during the same survey year were screened and excluded by duplicate reported telephone numbers. After the qualification screening and written informed consent from each of the eligible participants, face-to-face questionnaires were conducted anonymously in a private room by trained CDC staff. Supervisors are in charge of checking over the questionnaires and conducting supplementary surveys. After pre-test counseling and stringent aseptic procedures, skilled nurses drew 5 mL of venous blood from them. Throughout the 5 years of surveys, the venues, key interviewers, and recruitment strategies remained unchanged.

Data extracted in the questionnaire include demographic information, HIV knowledge, sexual behaviors in the past 6 months (P6M), recreational drug use, diagnosis of sexually transmitted diseases (STD), access to HIV prevention services, and HIV testing in the past 12 months (P12M). We provided rik-reduction counseling for each participant after the test. There was no collection of names or identifiers, however, cell phone numbers were recorded to inform laboratory test results and give referral services as needed. Confirmed HIV cases were subsequently referred to the ART clinic for treatment while current syphilis cases were referred to STD clinics for standard treatment. The receiving clinics will inform the CDC staff whether the case received the necessary treatment promptly.

### Specimen Collection and Laboratory Tests

All the blood samples were tested for HIV and syphilis. Laboratory testing was performed under the National Guideline for Detection of HIV/AIDS (13) and Diagnosis for syphilis (14). We used a rapid test (Determine HIV-1/2, Alere Medical, Chiba Prefecture, Japan) for preliminary HIV-1 antibody screening and an enzyme-linked immunoassay (HIV Ag/Ab ELISA KIT 96T, Zhuhai Livzon Diagnostics, China) for HIV retesting and a western blot assay (HIV BLOT 2.2, MP, Singapore) for HIV confirmation. We used both a treponema pallidum particle assay (TPPA) (Alere Medical) and a rapid plasma reagin test (Diagnosis; Shanghai, Kehua, China) to detect syphilis. Positive results on both tests were considered a confirmed current syphilis infection.

### Data Analysis

In this study, UAI was defined as failure to consistently use a condom during anal sex in the P6M. UAI was categorized as ‘No' if having no anal sex or always using condom every time and ‘Yes' if condoms were not used all the time. ‘Received any intervention' was defined as receiving any service including AIDS/STI educational materials distribution, condom distribution, lubricant distribution, peer education, HIV counseling, or HIV testing in the past 12 months. ‘Multiple sexual partners' was defined as having two or more male sexual partners in the P6M. Recreational drugs included poppers (alkyl nitrites), ecstasy, ice (methamphetamine), amphetamine, tramadol, and ketamine (8, 15).

Data were double entered using Epi Data (version 3.1, Denmark) and analyzed using SPSS (version 18.0, LEAD Technologies Inc.). Descriptive statistics were used to describe participants' demographic characteristics and prevalence rates. Linear-by-linear chi-square tests were performed to analyze trends in socio-demographic characteristics, HIV prevalence, risk behaviors, and HIV testing from 2016 to 2020. The univariate and multivariate logistic regression analyses were performed to analyze the factors associated with HIV infection. The variables with a *P*-value <0.1 in univariate analyses were included in the multivariate regression analysis. A stepwise multivariate logistic regression model was conducted to determine variables associated with HIV infection. All statistical significance test results were reported as *p*-values, which <0.05 (two-tailed) was considered statistically significant.

## Results

### Demographic and Social Characteristics

A total of 775 SMSM were recruited in our survey (220, 112, 171, 142, and 120, respectively) during the 5 years from 2016 to 2020. The socio-demographic characteristics of the participants were shown in Table 1. The average age of all the participants was (22.2±2.7). From 2016 to 2020, the average age was (22.2 ± 2.4), (22.5 ± 2.3), (21.5 ± 2.6), (22.9 ± 3.4), and (22.4 ± 2.6), respectively. The majority of them were Jiangsu residents, highly educated and homosexual. Among the 775 SMSM, 65.5% of them had lived in Nanjing city for more than 2 years and 83.7% of them had sought partners using internet or social networking applications. The composition of the participants and their corresponding characteristics were comparable in the five surveys except for venues of seeking partner (Table 1).

**Table 1:** Socio-demographic characteristics among SMSM in Nanjing, China, 2016–2020.

**Variables**	**2016 *n* (%)**	**2017 *n* (%)**	**2018 *n* (%)**	**2019 *n* (%)**	**2020 *n* (%)**	**Total *n* (%)**	**Linear-by -linear χ^2^**	***P*-value**
Number	220	112	171	142	120	775		
Registered permanent residence								
Jiangsu province	139 (66.2)	71 (63.4)	103 (60.2)	89 (62.7)	70 (58.3)	472 (60.9)	1.898	0.168
Outside Jiangsu province	71 (33.8)	41 (36.6)	68 (39.8)	53 (37.3)	50 (41.7)	283 (36.5)		
Living in Nanjing								
<2 years	62 (29.5)	34 (30.4)	68 (39.8)	38 (26.8)	45 (37.5)	247 (31.9)	1.069	0.301
≥2 years	148 (70.5)	78 (69.6)	103 (60.2)	104 (73.2)	75 (62.5)	508 (65.5)		
Education								
Senior middle school and lower	9 (4.3)	2 (1.8)	14 (8.2)	13 (9.2)	8 (6.7)	46 (5.9)	3.719	0.054
College and higher	201 (95.7)	110 (98.2)	157 (91.8)	129 (90.8)	112 (93.3)	709 (91.5)		
Sex orientation								
Homosexual	153 (72.8)	86 (76.8)	100 (58.5)	95 (66.9)	86 (71.7)	520 (67.1)	1.642	0.200
Bisexual	44 (21.0)	21 (18.8)	61 (35.7)	35 (24.6)	25 (20.8)	186 (24.0)		
Heterosexual/not sure	13 (6.2)	5 (4.5)	10 (5.8)	12 (8.5)	9 (7.5)	49 (6.3)		
Venues of seeking partners								
Bathroom/parks/public restrooms/clubs	39 (18.6)	21 (18.8)	23 (13.5)	18 (12.7)	5 (4.2)	106 (13.7)	13.365	<0.001
Internet/social networking applications	171 (81.4)	91 (81.3)	148 (86.5)	124 (87.3)	115 (95.8)	649 (83.7)		

### Sex Behaviors and HIV/ Syphilis Prevalence SMSM

**Figure 1:**
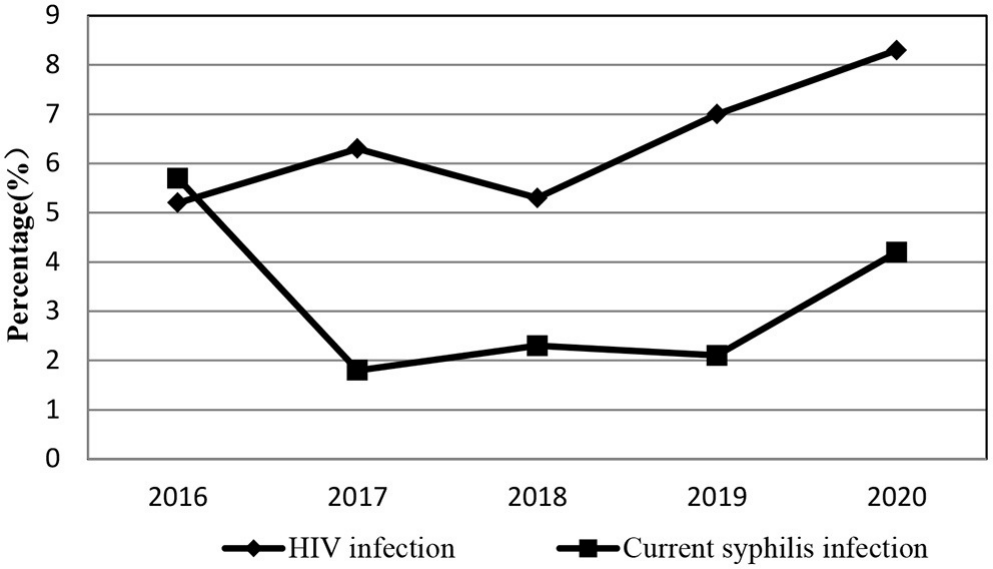
The trend in the prevalence of HIV/Syphilis among SMSM in Nanjing, China, 2016–2020.

The HIV prevalence among SMSM was 5.2, 6.3, 5.3, 7.0, and 8.3%, respectively. Although there seemed to be an increasing trend, the differences weren't statistically significant according to the linear-by-linear chi-square test (χ^2^ = 1.184, *P* = 0.277). Syphilis prevalence was 5.7, 1.8, 2.3, 2.1, and 4.2%, respectively. Despite the figures fell from 5.7percent in 2016 to 2.1percent in 2019 and rebounded to 4.2 percent in 2020, the differences were not statistically significant (χ^2^ = 0.999, *P* = 0.318). The trend of the prevalence of HIV/Syphilis was presented in Figure 1. As shown in Table 2, the proportion of consistently using condoms during anal intercourse (AI) increased significantly from 48.5 to 56.2%, whereas the proportion of having multiple male sex partners rose significantly from 46.2% in 2016 to 59.2% in 2020. The proportions of participants who had AI with a regular male partner demonstrated an increasing trend across the years (*P* < 0.05). In the meantime, the proportion of participants who tested for HIV in the P12M increased statistically (*P* < 0.05). The mean age at first AI with males was (19.4 ± 2.2) years old. The variables including AIDS awareness, age at first AI with males lower than 18, had AI, had AI with a casual male partner, had commercial anal sex, had vaginal sex, ever used recreational drugs, diagnosed with STD, and received any intervention showed no significant change over the 5 years.

**Table 2:** Trends in risk behavior and HIV testing among SMSM in Nanjing, China, 2016–2020.

**Variables**	**2016 *n* (%)**	**2017 *n* (%)**	**2018 *n* (%)**	**2019 *n* (%)**	**2020 *n* (%)**	**Total *n* (%)**	**Linear- by-line-ar χ^2^**	***P*-value**
AIDS awareness	184 (87.6)	102 (91.1)	150 (87.7)	131 (92.3)	113 (94.2)	680 (87.7)	4.126	0.389
Age at first AI with males <18 years	28 (13.3)	13 (11.6)	31 (18.1)	19 (13.4)	22 (18.3)	113 (14.6)	1.375	0.241
Multiple male sex partners in P6M	97 (46.2)	53 (47.3)	96 (56.1)	83 (58.5)	71 (59.2)	400 (51.6)	8.575	0.003
Had AI with males in P6M	167 (79.5)	89 (79.5)	143 (83.6)	115 (81)	105 (87.5)	619 (79.9)	2.733	0.098
Consistent condom use during AI in P6M*	81 (48.5)	43 (48.3)	79 (55.2)	71 (61.7)	59 (56.2)	333 (53.8)	4.243	0.039
Had AI with regular male partner in P6M	128 (61.0)	78 (69.6)	127 (74.3)	105 (73.9)	85 (70.8)	523 (67.5)	6.127	0.013
Had AI with casual male partner in P6M	116 (55.2)	59 (52.7)	116 (67.8)	80 (56.3)	70 (58.3)	441 (56.9)	0.735	0.391
Had commercial sex in P6M	4 (1.9)	2 (1.8)	9 (5.3)	4 (2.8)	2 (1.7)	21 (2.7)	0.113	0.737
Had vaginal intercourse in P6M	14 (6.7)	7 (6.3)	26 (15.2)	19 (13.4)	5 (4.2)	71 (9.2)	0.499	0.480
Ever used recreational drugs	64 (30.5)	18 (16.1)	37 (21.6)	33 (23.2)	28 (23.3)	180 (23.2)	1.607	0.205
Diagnosed with a STD in P12M	15 (7.1)	5 (4.5)	4 (2.3)	8 (5.6)	5 (4.2)	37 (4.8)	1.292	0.256
Received any intervention in P12M	152 (72.4)	75 (67.0)	130 (76.0)	109 (76.8)	89 (74.2)	555 (71.6)	1.108	0.293
Tested for HIV in P12M	107 (51.0)	56 (50.0)	85 (49.7)	87 (61.3)	71 (59.2)	406 (52.4)	4.093	0.043

AI, anal intercourse; P6M, past 6 months; P12M, past 12 months; STD, sexually transmitted diseases; * only surveyed those who had AI with males in P6M.

### Regression Analysis of HIV Infection Among SMSM

Factors associated with HIV infection among SMSM from the univariate analyses were presented in Table 3. Factors with *p*-values < 0.10 in univariate analyses were selected for the multivariate analysis. Factors associated with HIV infection from the multivariable logistic regression were reported in Table 4. Those who had UAI in the P6M (OR = 2.2, 95% CI: 1.1–4.0), ever used recreational drugs (*OR* = 2.3, 95%*CI*: 1.2–4.2) and currently syphilis infected (*OR* = 4.6, 95% *CI*: 1.7–12.9) were associated with higher risk for HIV infection. The participants who tested for HIV in the P12M (*OR* = 0.4, 95% *CI*: 0.2–0.8) were less likely to be infected with HIV compared with those not tested.

**Table 3:** Factors associated with HIV infection by univariate logistic regression analyses among SMSM in Nanjing, China.

**Factors**	**Number of subjects**	**HIV infection**	**HIV prevalence**	***OR* (95%*CI*)**	***P*-value**
Registered permanent residence					
Jiangsu province	472	31	6.6	1.0	
Outside Jiangsu province	283	16	5.7	0.9 (0.5–1.6)	0.615
Living in Nanjing					
<2 years	247	14	5.7	1.0	
≥2 years	508	33	6.5	1.2 (0.6–2.2)	0.659
Education					
Senior middle school and lower	46	4	8.7	1.0	
College and higher	709	43	6.1	0.7 (0.2–2.0)	0.477
Sex orientation					
Homosexual	520	30	5.8		
Bisexual	186	15	8.1	1.4 (0.8–2.7)	0.274
Heterosexual/not sure	49	2	4.1	0.7 (0.2–3.0)	0.626
Venues for meeting partners					
Bathroom/parks/public restrooms/clubs	106	7	6.6	1.0	
Internet/social networking applications	649	40	6.2	0.9 (0.4–2.1)	0.862
Awareness of AIDS knowledge					
No	75	6	8	1.0	
Yes	680	41	6	0.7 (0.3–1.8)	0.504
Role in homosexual intercourse					
Insertive	224	13	5.8	1.0	
Receptive	239	18	7.5	1.3 (0.6–2.8)	0.458
Versatile	292	16	5.5	0.9 (0.4–2.0)	0.874
Age at first anal intercourse with males				
≥18	642	35	10.6	1.0	
<18	113	12	5.5	2.1 (1.0–4.1)	0.040
AI with males in P6M					
No	136	7	5.1	1.0	
Yes	619	40	6.5	1.3 (0.6–2.9)	0.566
UAI in P6M					
No	469	20	4.3	1.0	
Yes	286	27	9.4	2.3 (1.3–4.3)	0.005
Multiple male sex partners in P6M					
No	355	16	4.5	1.0	
Yes	400	31	7.8	1.8 (1.0–3.3)	0.069
Ever used recreational drugs					
No	575	27	4.7	1.0	
Yes	180	20	11.1	2.5 (1.4-4.6)	0.003
Diagnosed with a STD in P12M					
No	718	42	5.8	1.0	
Yes	37	5	13.5	2.5 (0.9–6.8)	0.069
Received any intervention in P12M					
No	200	13	6.5	1.0	
Yes	555	34	6.1	0.9 (0.5–1.6)	0.650
Tested for HIV in P12M					
No	29	349	8.3	1.0	
Yes	18	406	4.4	0.5 (0.3–0.9)	0.030
Current syphilis infection					
No	729	41	5.6	1.0	
Yes	26	6	23.1	5.0 (1.9–13.2)	0.001

*AI, anal intercourse; UAI, unprotected anal intercourse; P6M, past 6 months; P12M, past 12 months; STD, sexually transmitted diseases*.

**Table 4:** Factors associated with HIV infection by multivariate logistic regression analysis among SMSM in Nanjing, China.

**Factors**	**β**	**SE**	**Wald χ2**	**Adjusted *OR* (95%*CI*)**	***P*-value**
UAI in P6M					
No				1.0	
Yes	0.770	0.313	6.054	2.2(1.2–4.0)	0.014
Ever used recreational drugs					
No				1.0	
Yes	0.818	0.321	6.497	2.3(1.2–4.2)	0.011
Tested for HIV in P12M					
No				1.0	
Yes	−0.805	0.319	6.356	0.4(0.2–0.8)	0.012
Current syphilis infection					
No				1.0	
Yes	1.528	0.524	8.522	4.6(1.7–12.9)	0.004

*UAI, unprotected anal intercourse; P6M, past 6 months; P12M, past 12 months*.

## Discussion

Our study found that HIV prevalence among SMSM in Nanjing remained steady from 2016 to 2020, coinciding with the stable HIV prevalence among general MSM during 2013−2017 (8). Although the syphilis prevalence fell from 2016 to 2019 and then rose somewhat in 2020, there is no discernible downward trend overall. The HIV prevalence among SMSM was still much lower than the general MSM population in Nanjing. Compared with other cities, HIV prevalence among SMSM in Nanjing was lower than the reports from seven cities of China (16) (6.5% between 2012 and 2013), Chongqing (17) (10.2% between 2013 and 2014), and Beijing (18) (7.5% between 2013 and 2014). It was also lower than HIV prevalence among young MSM in other countries [e.g., 7.8% in Chicago (19), 10.0% in in 21 US cities in 2008 (20), 24.0% in Thailand (21)]. However, we observed higher HIV prevalence than that in Changsha (22) (5.5% in 2018) and Tianjin (23) (3.15% in 2014 and 4.03% in 2016), which indicated that it was still a serious public health problem among SMSM in Nanjing.

We found an increasing trend in the proportion of consistent condom use during anal intercourse, addressing the concern as to the protective effect of condom use. Simultaneously, we also found an increasing trend in the proportion of HIV testing in P12M, which could attribute to the comprehensive prevention implementation among colleges in Nanjing. Since 2015, Nanjing was designated one of the demonstration zones and launched the “One Region, Different Strategies,” implementing a series of interventions such as HIV epidemic notification, trainers training action, peer education, promotion of condom use, and HIV testing in 51 colleges or vocational schools in Nanjing. The infection rate of HIV did not decrease due to the increase of condom use and detection frequency, indicating further combination prevention programs will be needed containing more evidence-based biomedical interventions besides behavioral and structural interventions to meet the current HIV prevention needs. WHO recommended Post-exposure prophylaxis (PEP) for preventing the acquisition of HIV in 2014 (24) and Pre-exposure prophylaxis (PrEP) in 2015 (25) as an additional prevention choice for people at substantial risk of HIV infection. Hence, we should add in-depth knowledge of PEP and PrEP in our propaganda and promote the critical vulnerable groups of students to obtain corresponding services to greatly impact on reducing the newly-infected cases as possible. Although slightly half of the participants had multiple sexual partners, lower than that reported in three cities of China (65.2%), alarming that the percentage of multiple sexual partners constantly rose from 46.2% in 2016 to 59.2% in 2020. Two factors may explain this result: First, smartphones and network apps can break through the time and place limits and facilitate real-time location to seek sexual partners conveniently using geospatial information technology (e.g. Blued, Aloha, Jack'd, and Zank). App-using MSM were more likely to have multiple sexual partners (26, 27); Second, the proportion of seeking partners via internet/social network apps showed a growing trend during the 5 years. In light of the severe HIV epidemic situation among SMSM in Nanjing, multiple sexual partners could increase the odds of HIV infection and transmission (5, 28). The new generation is not interested in stereotyped propaganda and traditional ideas, so the government must innovate targeted health education and intervention to prevent high-risk sexual behavior based on new media among the subgroup.

Our study showed that UAI in P6M, recreational drug use, HIV testing in P12M and current syphilis infection were associated with HIV infection among SMSM. Similar to other studies, UAI, recreational drug use, and current syphilis infection were the pushers of HIV infection in among SMSM (5, 23). Although closed to 90 percent of participants had sufficient HIV prevention knowledge, only less than half of them used condom consistently. It showed that SMSM had high-risk sexual behaviors and a separation between knowledge and behavior. Syphilis can facilitate HIV transmission because syphilis lesions increase the sexual transmission efficiency of HIV (29). Our study found the total prevalence was 3.2%, slightly lower than that reported among SMSM in Beijing (30) (3.6%) and seven major Chinese cities (5) (4.1%). However, nearly 5% of SMSM reported that they had been diagnosed with STD in the last year, indicating that syphilis and other STDs in SMSM should not be ignored. Therefore, it is imperative to strengthen sexual health and sexual morality education among youth and adolescents. Meanwhile, standard STD treatment and expanding HIV testing for STD outpatient patients need to be further implemented.

In recent years, new drug abuse has become a public health and social problem of global concern. Our study showed that almost a quarter of participants ever used recreational drugs. Although the proportion was lower than that reported among MSM in Beijing (26.8%) (31), two cities of China (32) (29.8%), Ireland (33) (36.0%), New Zealand (34) (36.7%), and the United Kingdom (35) (28.0%), we hadn't observed a declining trend during our study period. Rush popper, as a most common recreational drug, was widely used among MSM to help enhance sexual pleasure by dilating capillaries and relaxing anal sphincters, thus, reducing pain associated with anal sex (31). It is not legally restricted in China and can be purchased easily and cheaply through the internet with advanced internet. Two studies found a higher proportion of recreational drug use among people younger than 25 years old and high educated (36, 37), suggesting that warning education about recreational drugs and interventions addressing reducing recreational drug use also should be focused on SMSM. Furthermore, effective laws and special campaigns to crack down on recreational drug use should be developed, implemented and enforced.

HIV testing is an essential component of HIV prevention. Our study confirmed that HIV testing was a protective factor associated with HIV infection. The counseling that accompanies HIV testing could convey more knowledge or inspire motivation for HIV prevention and enhance HIV awareness, thus HIV testers might be more attentive to taking precautions by reducing high-risk behaviors to lower their HIV risk (18). Our study also showed that the HIV testing rate was over a half among SMSM, lower than that reported in 3 cities of China (70.6%) (38) and USA (78%) (39). HIV testing is central to the UNAIDS 90-90-90 Targets by 2020. However, there was still a large gap between HIV testing rate among SMSM in Nanjing and ‘UNAIDS 90% HIV detection target'. Compared with general MSM, there are additional barriers to HIV testing among students, including low-risk perception, fear of positive diagnosis, concern of disclosure of sexuality, and limited access to testing resources (40, 41). Previous studies suggested HIV self-testing can promote active detection, eliminate the concern of privacy disclosure, and improve the accessibility and pertinence of HIV testing among MSM in China (42, 43). Therefore, we should further expand HIV testing, especially HIV self-testing, promote the habit of regular detection and improve online post-test services in college students to ensure early detection and treatment of student HIV case, thus curbing the rapidly rising HIV epidemic in colleges and universities.

This study had several limitations. First, five cross-sectional studies were inherently descriptive and did not illustrate the causal relationship. Second, the participants were recruited from MSM venues, some MSM social platforms, and a government internet site and they may not be representative of MSM who do not go to these venues or visit the website, which may result in lack of information from hidden SMSM. Third, the questionnaire was answered based on subjective reports and MSM and HIV/AIDS are sensitive issues, leading to a possible information bias. Fourth, because the objects were MSM students in colleges or universities who were very difficult to contact, our study required participants to come to our VCT clinics for HIV testing and questionnaire, which formed a slightly smaller sample size per year.

## Conclusions

We observed stable HIV prevalence, increasing consistent condom use and increasing HIV testing rate among SMSM. However, an increase of multiple male partners and steady recreational drug use were observed. In response to the high HIV burden among SMSM in Nanjing, original comprehensive intervention measures, especially promoting condom use, expanding HIV testing and timely treatment, reducing recreational drug use, expanding STD screening and standard treatment should be continuously strengthened. To satisfy the current HIV prevention requirements among SMSM, new biological interventions like PEP and PrEP should be introduced and carried out as major components of combination prevention programs.

## Data Availability Statement

The raw data supporting the conclusions of this article will be made available by the authors, without undue reservation.

## Ethics Statement

The studies involving human participants were reviewed and approved by the Ethics Committee of the Nanjing National Center for AIDS and STD Control. Written informed consent to participate in this study was provided by the participants' legal guardian/next of kin.

## Author Contributions

SW, XL, and HS were contributed to data collection. WX and MQ contributed to laboratory testing, JD contributed to quality control, YX and XF were responsible for data analysis and manuscript writing, ZZ contributed to the study design and manuscript revision. All authors read and approved the final manuscript.

## Funding

This study was supported by Nanjing Municipal Key Medical Science and Technology Development Project (Grant no. ZKX19050) and Nanjing Key Medical Specialty Project of Infectious Diseases. The funding had no role in study design, data collection, but had a role in data analysis and preparation for manuscript.

## Conflict of Interest

The authors declare that the research was conducted in the absence of any commercial or financial relationships that could be construed as a potential conflict of interest.

## Publisher's Note

All claims expressed in this article are solely those of the authors and do not necessarily represent those of their affiliated organizations, or those of the publisher, the editors and the reviewers. Any product that may be evaluated in this article, or claim that may be made by its manufacturer, is not guaranteed or endorsed by the publisher.
